# Design of a biocatalytic cascade for the enzymatic sulfation of unsulfated chondroitin with *in situ* generation of PAPS

**DOI:** 10.3389/fbioe.2023.1099924

**Published:** 2023-01-16

**Authors:** Dianelis T. Monterrey, Raúl Benito-Arenas, Julia Revuelta, Eduardo García-Junceda

**Affiliations:** Department Bio-Organic Chemistry, BioGlycoChem Group, Instituto de Química Orgánica General (IQOG-CSIC), Spanish National Research Council, Madrid, Spain

**Keywords:** sulfotransferases, PAPS synthase, ATP sulfurylase, APS kinase, chondroitin sulfate, glycosaminoglycans, biocatalytic cascade, enzymatic sulfation

## Abstract

Sulfation of molecules in living organisms is a process that plays a key role in their functionality. In mammals, the sulfation of polysaccharides (glycosaminoglycans) that form the proteoglycans present in the extracellular matrix is particularly important. These polysaccharides, through their degree and sulfation pattern, are involved in a variety of biological events as signal modulators in communication processes between the cell and its environment. Because of this great biological importance, there is a growing interest in the development of efficient and sustainable sulfation processes, such as those based on the use of sulfotransferase enzymes. These enzymes have the disadvantage of being 3′-phosphoadenosine 5′-phosphosulfate (PAPS) dependent, which is expensive and difficult to obtain. In the present study, a modular multienzyme system was developed to allow the *in situ* synthesis of PAPS and its coupling to a chondroitin sulfation system. For this purpose, the bifunctional enzyme PAPS synthase 1 (PAPSS1) from *Homo sapiens*, which contains the ATP sulfurylase and APS kinase activities in a single protein, and the enzyme chondroitin 4-*O*-sulfotransferase (C4ST-1) from *Rattus norvegicus* were overexpressed in *E. coli*. The product formed after coupling of the PAPS generation system and the chondroitin sulfation module was analyzed by NMR.

## 1 Introduction

In living beings there are many sulfated compounds, including proteins, steroids, polysaccharides, and different metabolites. Sulfation is an essential biological process for regulating the bioactivity of these molecules (Günal et al., 2019). These sulfated molecules perform different functions in different organisms; embryogenesis, inflammation, signal transduction, and coagulation are some of the most representative examples of their functions (Chapman et al., 2004). In mammals, the sulfation of polysaccharides (glycosaminoglycans) that form the proteoglycans present in the extracellular matrix is particularly important (Bishop et al., 2007; Costa et al., 2017). Among the most relevant functions of proteoglycans, their role as modulators of signals in communication processes between the cell and its environment stands out (Bülow et al., 2008; Mikami and Kitagawa, 2017; Chhabra et al., 2021). These properties are mainly associated with the carbohydrate chain of the glycosaminoglycan (chondroitin and heparan sulfate, among others) and result precisely from the negative charges they have, as well as from their density and position (Holt and Dickson, 2005; Gama et al., 2006; Sakamoto et al., 2019; Lin et al., 2020). The sulfated residues of glycosaminoglycans interact with growth factors, cytokines, morphogenetic proteins, enzymes, and inhibitory or stabilizing glycoproteins of the pericellular or extracellular matrix. These interactions have various effects on cell metabolism, cell differentiation, matrix synthesis and stabilization, and remodeling of the developing tissue. Therefore, they are essential for cellular control of homeostasis (Hayes et al., 2018).

Extraction of these polysaccharides from natural sources is excessively expensive in many cases. Chemical sulfation is a complex process involving multiple steps and protection and deprotection processes that often result in loss of regio- and chemoselectivity. In addition, hazardous chemical reagents such as sulfur trioxide pyridine, sulfuric acid, and dicyclohexylcarbodiimide are used in the process (Simpson and Widlanski, 2006). For this reason, synthetic alternatives in the context of Green Chemistry, such as the use of enzymes, are of particular importance (García-Junceda et al., 2004). Sulfotransferases (EC. 2.8.2.5) are the enzymes that carry out the sulfation reaction in living organisms and catalyze the transfer of the sulfate residue of 3′-adenosine 5′-phosphosulfate (PAPS) to hydroxyl and amine groups from a variety of acceptors (Chapman et al., 2004). Sulfotransferases can be divided into two main classes: 1) those that are found free in the cytoplasm and mainly catalyze the sulfation of small molecules, such as hormones or xenobiotic compounds (Ayuso-Fernández et al., 2014), and 2) membrane-associated sulfotransferases whose substrates are larger biomolecules, such as carbohydrates and proteins (Chapman et al., 2004).

**FIGURE 1:**
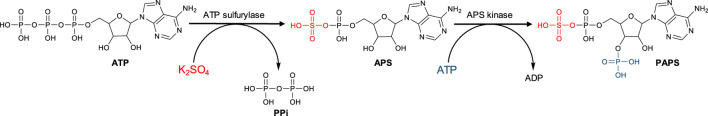
Schematic representation of the PAPS synthesis.

Industrial application of sulfotransferases in sulfation processes has been limited due to the need to use PAPS as donor substrate, a compound with high cost (400 €/mg), low bioavailability and stability. In addition, yield is limited because the PAP (3′-phosphoadenosine 5′-phosphate) formed after sulfation inhibits sulfotransferase even at micromolar concentrations. To overcome these limitations, PAPS can be regenerated (Burkart et al., 1999; Burkart et al., 2000; Chen et al., 2007; Xu et al., 2008; Peterson et al., 2009; Xu et al., 2011; Wang et al., 2020) or formed *in situ* from AMP and inorganic sulfate (Lin et al., 1995; Xu et al., 2011; Zhou et al., 2011; Xu et al., 2014; Bao et al., 2015; An et al., 2017). Synthesis of PAPS is carried out from sulfate and two molecules of ATP by two successive enzymatic activities (Figure 1): ATP sulfurylase, which catalyzes the formation of adenosine 5′-phosphosulfate (APS) from ATP and sulfate; and an APS kinase, which catalyzes the phosphorylation of APS to form PAPS, consuming second ATP molecule (Datta et al., 2020).

The synthesis of PAPS is a multi-step process that involves the activation of inorganic sulfate, which is a very stable oxyanion that usually resists gentle attempts to make it undergo chemical bonding (Mueller and Shafqat, 2013). The first step involves the formation of adenosine 5′-phosphosulfate (APS) from ATP and inorganic sulfate, catalyzed by ATP sulfurylase (EC 2.7.7.4), which transfers the AMP residue from ATP to sulfate and simultaneously releases pyrophosphate. This reaction leads to the formation of a high-energy phosphoric acid-sulfuric acid-anhydride bond, which is the chemical basis for sulfate activation (Leyh, 1993). In the second step, PAPS is then formed after phosphorylation of APS catalyzed by an APS kinase (EC 2.7.1.25) and consumption of a second ATP molecule (Lansdon et al., 2004). These two enzymatic activities are located in two independent proteins in bacteria, yeasts, fungi, and plants (Leustek et al., 1994; Borges-Walmsley et al., 1995). In humans, however, they are co-located in a bifunctional protein called phosphoadenosine phosphosulfate synthase (PAPSS1) (Venkatachalam et al., 1998; Harjes et al., 2004).

Herein, in this work the development of a bimodular biocatalytic system that allows the generation of PAPS for subsequent use in the sulfation of compounds of interest is described. For the development of the PAPS generation module, the bifunctional enzyme PAPSS1 from *Homo sapiens* was overexpressed in *E. coli*. In the sulfation module, sulfation of unsulfated chondroitin was performed using the enzyme C4ST-1 from *R. norvegicus* as proof of concept. This enzyme is an example of a membrane-associated sulfotransferase and catalyzes the transfer of a sulfate group to position 4 of the *N*-acetyl-d-galactosamine (GalNAc) residues of chondroitin polysaccharides or non-sulfated dermatan (Yamauchi et al., 1999; Mikami et al., 2003). This enzyme is quite specific for the acceptor substrate, since only unsulfated CS and dermatan sulfate behave as good substrates, whereas when CS A and CS C were used as acceptors, the observed activity was much lower (Yamauchi et al., 1999; Mikami et al., 2003). However, when dermatan sulfate, keratan sulfate, CS E, heparan sulfate or heparin were tested as substrates, no activity was observed (Yamauchi et al., 1999). On the other hand, C4ST-1 preferentially sulfates position 4 of GalNAc residues flanked by GlcUA residues, both on the reducing and non-reducing ends (Yamauchi et al., 1999; Mikami et al., 2003).

## 2 Materials and methods

### 2.1 Materials

Synthesis of the genes for the enzymes PAPS synthase 1 and sulfotransferase C4ST-1 and their subsequent cloning into plasmids pET-28b(+) and pET-32b(+), respectively, was performed by GenScriptTM (Piscataway, NJ). The original nucleotide sequence of both enzymes was optimized to implement their expression in *E. coli* (OptimumGene™ algorithm). *E. coli* strain BL21 (DE3) was obtained from Promega Biotech Ibérica S.L. (Madrid, Spain). Plasmid purification was performed using the GenElute™ Plasmid Miniprep Kit from Sigma-Aldrich (Darmstadt, Germany). Promega 1 kb DNA Step Ladder Molecular Weight Markers and restriction enzymes were purchased from ThermoFisher Scientific Inc. (Waltham, MA). Kanamycin, pyruvate kinase/lactic dehydrogenase mixture, pyrophosphatase, DNase I, and other reagents were purchased from Sigma-Aldrich (Darmstadt, Germany). Isopropyl-β-d-thiogalactopyranoside (IPTG) was purchased from Applichem GmBH (Darmstadt, Germany). Imidazole and NADH were purchased from Thermo Fisher Scientific Inc. (Waltham, MA). Bradford reagent and acrylamide/bis-acrylamide solution of 30% (29:1) used for protein analysis by SDS-PAGE were purchased from Bio-Rad (Hercules, CA). The Low Molecular Weight Calibration Kit from GE Healthcare was used. SDS-PAGE Gels were run on a MiniProtean^®^ Tetracell cuvette from Bio-Rad (Hercules, CA). Agarose gels were run in a RunOne™ Electrophoresis Cell cuvette from EmbiTec (San Diego, CA) using SYBR Safe dye from ThermoFisher Scientific (Waltham, MA) for staining DNA in electrophoresis gels. Densitometric analysis of acrylamide genes was performed using a Gene Flash Bio Imaging Photodocumenter from Syngene Ltd. (Bengaluru, Karnataka) and GeneTools 3.07 software. Spectrophotometric assays were carried out in a UV-Visible SPECTRAmax-384 PLUS, from Molecular Devices, LLC (San José, CA). Iminodiacetic acid agarose (IDA-agarose) was purchased from Agarose Bead Technologies (Miami, FL). Solvents were of analytical grade. DNA manipulation was performed according to standard procedures (Sambrook et al., 1989). Unsulfated chondroitin (CS-0S) was chemically prepared following the procedure previously described in Benito-Arenas et al. (2018). ^1^H-NMR was performed at room temperature using deuterated water (D_2_O) as solvent in a Varian INOVA-500 (^1^H 500 MHz) instrument. Chemical shift values are given in parts per million (δ, ppm). In addition, the two-dimensional NMR experiment Heteronuclear Single Quantum Correlation (HSQC) was performed to further confirm the signal assignments. The MestReNova v.11.0.1-17801 program was used to process all NMR spectra, both one-dimensional and two-dimensional.

### 2.2 Heterologous expression and purification of recombinant human PAPSS1 enzyme

Cloning of *papss*1 gene was performed in the expression plasmid pET-28b(+), which allows induction of protein overexpression with IPTG. In addition, the protein is expressed fused to a 6-His tag at the *N*-terminal end, allowing its one-step purification by ion metal affinity chromatography (IMAC) from cell-free extract (CFE). The plasmid, designated pET-28b(+)-*papss1*, was then transformed into electrocompetent *E. coli* BL21 (DE3) cells. The transformed cells were growth in LB agar plates containing 30 μg/ml of the antibiotic kanamycin. The plates were kept overnight at 37°C. Three randomly selected colonies were grown in 5 ml LB containing kanamycin (30 μg/mL) for plasmid purification. The purified plasmids were subjected to a double digestion with the restriction enzymes *Eco*RI and *Hin*dIII in a reaction volume of 10 μl according to the protocol specified by the manufacturer. The different DNA fragments were analyzed by electrophoresis in .8% agarose gel in TAE (40 mM Tris-acetate, 1 mM EDTA, pH 8.0). Visualization of the gel with SYBR Safe dye revealed two bands (Supplementary Figure S1): a band of 5,368 bp corresponding to plasmid pET-28-b(+) and a band of 1,872 bp corresponding to the expected size of the *papss1* gene.

For PAPSS1 enzyme expression, preinocula of recombinant colonies were prepared in 5 ml LB medium containing 30 μg/ml kanamycin and incubated overnight at 37°C with shaking (160 rpm). The culture was extended and incubated under the same conditions until the exponential phase (OD_600nm_ = .5–.7) was reached. At this point, protein expression was induced with IPTG at a final concentration of 1 mM. Orbital agitation was kept constant, and the temperature was lowered to 30°C to avoid the formation of inclusion bodies. The culture was then centrifuged at 2,500 × g for 20 min at 4°C to collect the cells. The pellet was resuspended in Na_2_HPO_4_ buffer (50 mM, 300 mM NaCl, pH = 8.0). Cell disruption was performed by sonication (70% amplitude, 5 s pulse and 20 s pause between pulses). The lysed cells were separated by centrifugation of the mixture at 8,000 rpm for 20 min. The supernatant obtained was treated with DNaseI (10 μg/ml cells) and MgCl_2_ (.95 μg/ml cells) on ice for 20 min. Streptomycin (1% wt/vol) was then added and allowed to act for an additional 20 min to precipitate the resulting nucleotides. Finally, the cell-free extract (CFE) containing the soluble protein fraction (separated from precipitates and inclusion bodies) was obtained by centrifugation at 8,000 rpm for 20 min at 4°C.

Purification of the enzyme was carried out according to two strategies. First, it was performed by affinity chromatography with divalent metals (IMAC) using high-density Co_2_^+^-IDA-agarose resin. The resin was packed into a propylene chromatography column using a suitable column filter. The resin was washed and equilibrated with 50 mM Na_2_HPO_4_, 300 mM NaCl, 10 mM imidazole, pH 8.0 buffer. The CFE was added to the resin at a ratio of 1:1 (v/v). The resin was washed with 3 volumes of the same buffer to eliminate non-specific binding proteins. Finally, the protein was eluted with a column volume of 50 mM Na_2_HPO_4_, 300 mM NaCl, 2 M imidazole, pH 8.0. Subsequently, the imidazole was removed from the medium by ultrafiltration so that it was exchanged for 50 mM Na_2_HPO_4_ pH 8.0 buffer. To achieve an even higher degree of purity, enzyme purification by size exclusion chromatography was performed. HiLoad 26/60 Superdex 200 PG size exclusion columns were used, controlled by a AKTA-FPLC system from GE Healthcare Life Science. 50 mM Na_2_HPO_4_ and 150 mM NaCl buffer at pH 7.2 with an isocratic flow of 1.0 ml/min were used as mobile phase. Fractions containing PAPSS1 were pooled and concentrated. All fractions were analyzed by SDS-PAGE on 11% acrylamide/bis-acrylamide gels and by spectrometric activity and protein concentration determination.

### 2.3 Analysis of the enzymatic activity of the recombinant PAPSS1

**FIGURE 2:**
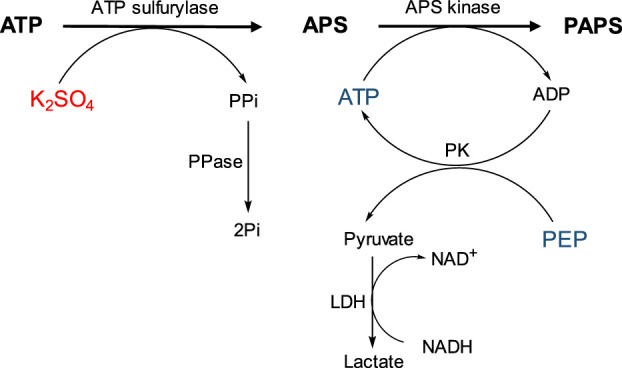
Schematic representation of the coupled spectrophotometric assay that allows measurement of PAPSS1 activity by the concomitant oxidation of NADH.

The activity of recombinant PAPSS1 was measured using a PK/LDH-coupled spectrophotometric assay that allows monitoring of ADP generated during APS phosphorylation by the concomitant oxidation of NADH. This assay allows not only spectrophotometric monitoring of the reaction, but also regeneration of ATP consumed by APS kinase, thus avoiding its inhibition by ADP. The enzyme pyruvate kinase (PK) catalyzes the transfer of a phosphate group from phosphoenolpyruvate (PEP) to ADP formed in the APS phosphorylation reaction. At the same time, the pyruvate formed is reduced to lactate by LDH consuming NADH, which can be measured spectrophotometrically (Figure 2).

Reactions were carried out in 1 ml of Tris-HCl buffer (50 mM pH = 8.0) containing ATP (12.5 μmol), K_2_SO_4_ (15 μmol), MgCl_2_ (25 μmol), PEP (1.0 μmol), NADH (.3 μmol), and the enzymes LDH (5 μg/ml), PK (5 μg/ml), and PAPSS1 (126.5 μg/ml). Because of the strong inhibition of ATP sulfurylase activity by PPi, PPase (10 μg/ml) was also added to degrade the pyrophosphate formed (Figure 2). In parallel, reactions were monitored by HPLC using a Dionex PDA-100 chromatograph with a C18 Discovery column (250 mm × 4.6 mm; Ø = 5 μm). The mobile phase used was 20 mM Na_2_HPO_4_ buffer pH 6.0 with isocratic flow .5 ml/min and detection at 260 nm. Initially, the separation method was optimized using standards for ATP and ADP (Supplementary Figure S2). Reactions were monitored for 18 h, with aliquots taken at 15 min, 30 min, and 18 h. As a control, similar reactions were analyzed but without the ATP regeneration system (PK).

### 2.4 Heterologous expression and purification of recombinant chondroitin-4-*O*-sulfotransferase (C4ST-1) from *Rattus norvegicus*

For the cloning of the C4ST-1 enzyme from *R. norvegicus*, the procedure described by He et al. (2017) for the cloning of the human enzyme was followed as the sequence of the *R. norvegicus* enzyme shows 96.88% similarity to the human C4ST-1 sequence. The first 60 amino acids of the C4ST-1 protein (1Met-60Leu) were removed because they have been identified as a putative transmembrane domain (He et al., 2017). The *chst11* gene modified in this way was cloned between the restriction enzymes *Bam*HI and *Xho*I of the plasmid pET-32b(+). This plasmid allows the N-terminal end of C4ST-1 to be fused to TrxA to increase the solubility of the recombinant protein, and additionally to a 6-histidine peptide to allow its purification by IMAC chromatography. The resulting pET-TrxA-*chst11* plasmid was transformed into competent *E. coli* BL21 (DE3) cells which were incubated on LB-agar plates with 250 μg/ml ampicillin. The plates were kept at 37°C overnight. Three randomly selected colonies were grown in 5 ml of LB and ampicillin (250 μg/ml) to purify their plasmids and analyzed by double digestion with the restriction enzymes *Bam*HI and *Xho*I in a reaction volume of 10 μl according to the protocol indicated by the manufacturer. The different DNA fragments were analyzed by electrophoresis in agarose gel at .8% in TAE (Tris-acetate 40 mM, EDTA 1mM, pH = 8.0). Two bands were observed in each of the colonies (Supplementary Figure S3): one of the bands, with an approximate size of 6,000 bp, corresponds to the plasmid pET32b(+); the other band, with an approximate size of 900 bp, is consistent with the expected size for the *chst11* gene encoding the C4ST-1 protein (892 bp).

Overexpression of the C4ST-1 enzyme was performed from a colony containing the *chst11* gene. The clone was incubated at 37°C with shaking at 160 rpm in LB medium containing 250 μg/ml ampicillin until an OD_600nm_ between .5–.7 was reached. At this point, expression was induced with .2 mM IPTG and the temperature was reduced to 22°C with the culture shaken at 160 rpm all night. Protein extraction followed the same steps described above for the PAPSS1 enzyme until the cell-free extract was obtained.

### 2.5 Coupling of the PAPS generation module and the sulfation module

The coupling of the PAPS generation module and the sulfation module was performed using a desulfated chondroitin (CS-0S) previously obtained in our laboratory as a substrate to be sulfated (Benito-Arenas et al., 2018). The PAPSS1 enzyme was used after purification by IMAC, whereas the C4ST-1 enzyme was used without purification. For the PAPS generation module, ATP (12.5 μmols), K_2_SO_4_ (15 μmols), MgCl_2_ (25 μmols), PEP (1 μmol), NADH (.3 μmols), and LDH enzymes (5 μg/ml, PK (5 μg/ml), pyrophosphatase (10 μg/ml), and PAPSS1 (126.5 μg/ml) were used. The sulfation module consisted of 6.0 mg/ml CS-0S and a volume of 25 μl of CFE of enzyme C4ST-1. The reaction was performed in 1 ml of 50 mM Tris-HCl buffer at pH = 8.0. The reaction was maintained for 72 h with orbital shaking at room temperature. Purification of the product of coupling the two modules was performed by dialysis against distilled water using a membrane with a cut-off of 3.5 KDa for 24 h. The resulting solution was lyophilized to give the product as a white solid. The product was identified by proton nuclear magnetic resonance (^1^H-NMR) experiments at room temperature using deuterated water (D_2_O) as solvent and two-dimensional heteronuclear single quantum correlation (HSQC) NMR experiments. Chemical shift values were compared to those previously described for authentic samples of the product (Benito-Arenas et al., 2018).

## 3 Results and discussion

### 3.1 Heterologous expression and purification of the recombinant enzyme PAPSS1

**FIGURE 3:**
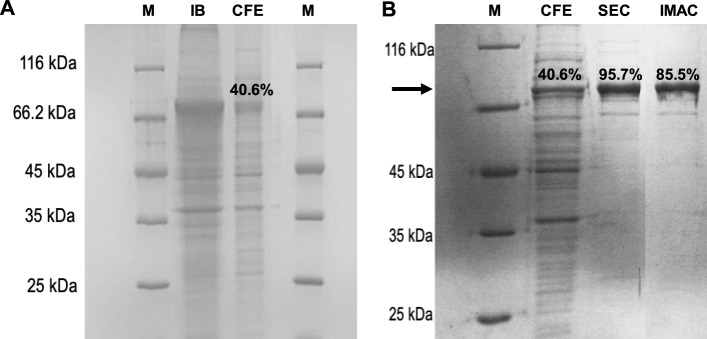
SDS-PAGE analysis of **(A)** PAPSS1 overexpression in transformed *E. coli* BL21 (DE3) cells: M, molecular weight marker; IB: insoluble protein fraction (inclusion body), CFE, soluble protein fraction, and **(B)** PAPSS1 purification by IMAC and size exclusion chromatography (SEC).

When PAPSS1 protein overexpression was analysed, the appearance of a band with a molecular mass of approximately 72 kDa was observed, which corresponds to the expected mass of the PAPSS1 protein. Although a large fraction of the recombinant protein is insolubly expressed in the form of inclusion bodies, PAPSS1 in the soluble fraction accounts for 40.6% of the total proteins present, making it the major protein (Figure 3A). As mentioned previously, PAPSS1 was purified using two alternative chromatographic techniques: IMAC and size exclusion chromatography. A high degree of purity was achieved with both techniques. IMAC achieved a purity of 85.5%, whereas size exclusion chromatography yielded an even higher purity of 95.7% (Figure 3B). Although better results were obtained with size exclusion chromatography than with IMAC, the latter technique was used for successive purifications of protein from CFE. This was mainly because of the simplicity of the method and the shorter time required to obtain the pure protein compared with size exclusion chromatography. In addition, no non-specific interactions were observed for the remaining 14.5% of the protein in reactions performed with IMAC-purified PAPSS1.

### 3.2 Analysis of PAPSS1 activity and ATP regeneration

**FIGURE 4:**
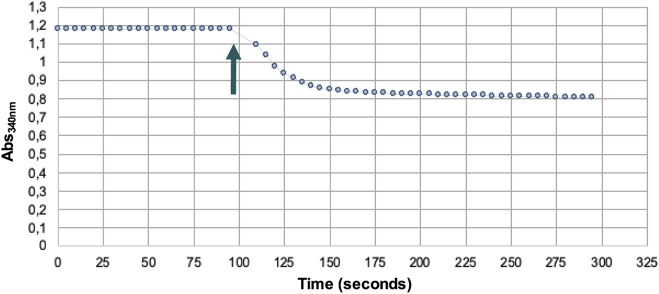
Spectrophotometric assay of PAPSS1 activity. Arrow indicates the addition of the substrate K_2_SO_4_.

The activity of PAPSS1 was monitored spectrophotometrically thanks to the coupling of the PK/LDH system, which also allows the regeneration of ATP used for the phosphorylation of APS catalyzed by the APS kinase activity of PAPSS1. The reaction was initiated by adding the K_2_SO_4_ substrate to the reaction medium (Figure 4).

**FIGURE 5:**
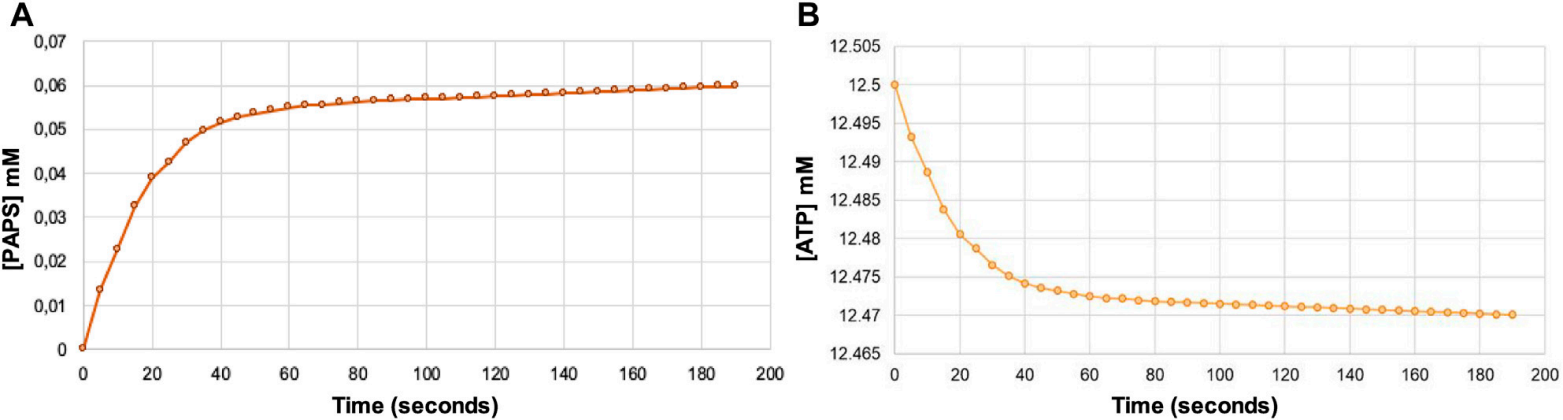
Indirect estimate of the formation of PAPS **(A)** and the concomitant ATP consumption **(B)** over reaction time from the decrease in absorbance at 340 nm due to NADH oxidation using the Lambert-Beer law. Note that the formation of one PAPS molecule requires the consumption of two ATP molecules.

The decrease in absorbance at 340 nm due to the oxidation of NADH allowed us to indirectly determine the amount of PAPS formed. In this way, it was determined that .06 μmol of PAPS was formed (Figure 5A), corresponding to a yield of 4.8%, whereas only .03 μmol of ATP was consumed (Figure 5B). These results indicate that regeneration of ATP consumed during phosphorylation of APS occurs, thus regenerating at least one of the two equivalents used in the synthesis of PAPS.

**FIGURE 6:**
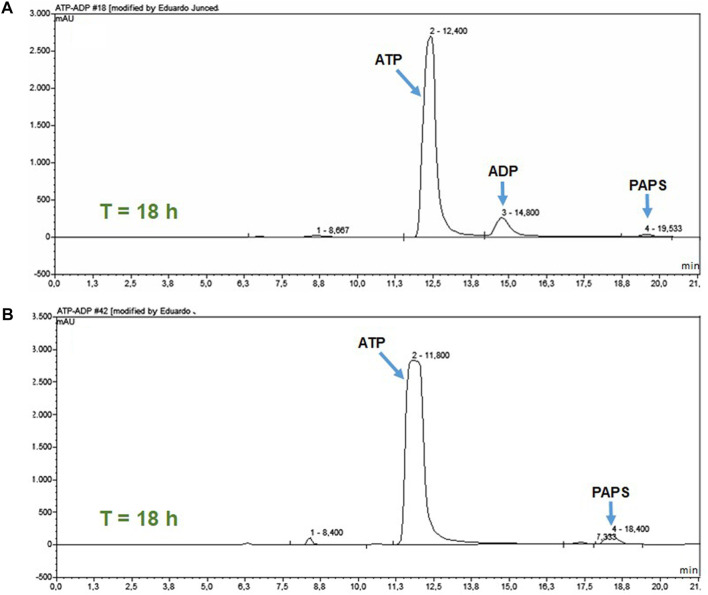
HPLC analysis of the ATP regeneration module at 18 h of reaction. **(A)** PAPS synthesis reaction without the coupled regeneration system. **(B)** Reaction with the coupled ATP regeneration module.

To analyze the effect of ATP regeneration on PAPSS1-catalyzed PAPS synthesis, two reactions of PAPS synthesis were followed for 18 h. In one of the reactions, the ATP regeneration system was coupled (Figure 2), whereas in the other this system was eliminated. Since this second reaction could not be analyzed spectrophotometrically, both reactions were analyzed by HPLC (Figure 6). In the reaction in which the ATP regeneration module was eliminated (Figure 6A), a peak with a retention time of 14.8 min appears, corresponding to the retention time of the ADP pattern, and the peak corresponding to PAPS is practically missing. On the other hand, in the chromatogram of the reaction with the coupled ATP regeneration system (Figure 6B), the appearance of the peak corresponding to ADP was not observed and the peak corresponding to PAPS increased significantly.

These results confirm that the PK/LDH system is efficient in ATP regeneration and, furthermore, that this regeneration is necessary for the synthesis of PAPS because the APS kinase activity of the PAPSS 1 enzyme is strongly inhibited competitively by APS (Yanagisawa et al., 1998; Harjes et al., 2004; Lansdon et al., 2004; Harjes et al., 2005; Sekulic et al., 2007; Mueller and Shafqat, 2013). This inhibition occurs through the formation of a dead-end complex of the enzyme with the APS and the ADP resulting from the phosphorylation reaction. The formation of this complex is favored by the fact that the affinity of the enzyme-ADP complex for APS is greater than that showed by the apoenzyme (Mueller and Shafqat, 2013).

### 3.3 Heterologous expression of the recombinant enzyme C4ST-1

**FIGURE 7:**
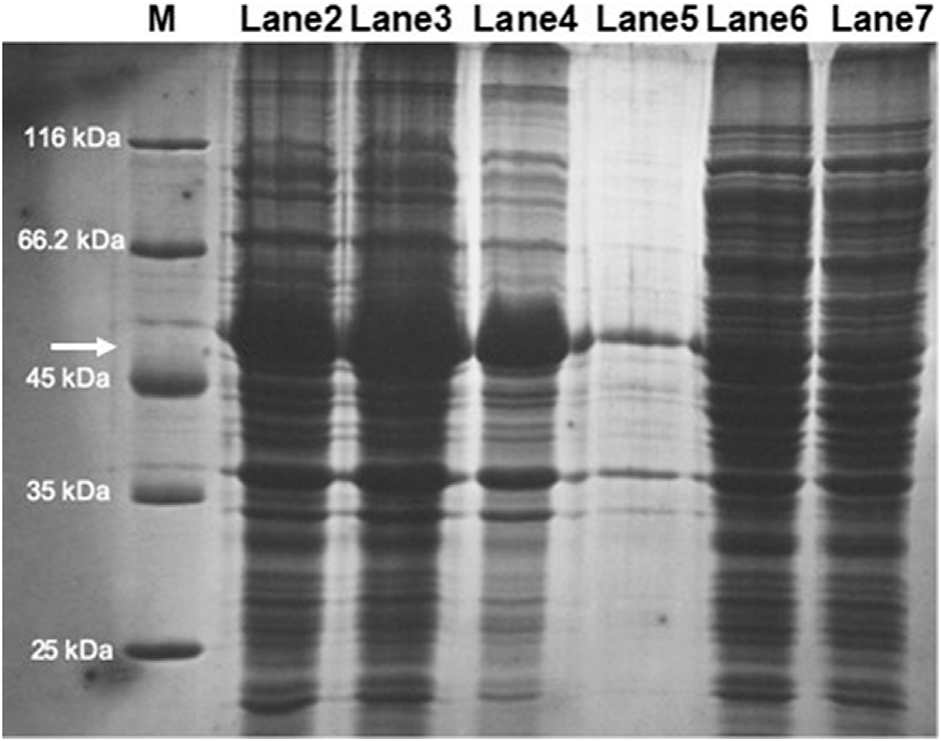
SDS-PAGE analysis of C4ST-1 overexpression in transformed *E. coli* BL21 (DE3) cells: M, molecular weight marker; Lanes 2–4: insoluble protein fraction (IPF); Lanes 5–7: soluble protein fraction (SPF). The arrow points to the protein whose size matches that of C4ST-1.

SDS-PAGE analysis of the soluble and insoluble fractions of the cell-free extract obtained from the *E. coli* strain transformed with the plasmid pET32b(+)-chst11 revealed a major protein in the insoluble fraction with a molecular mass of approximately 50 kDa, consistent with the size previously estimated for the C4ST-1 enzyme (54 kDa). Unfortunately, the majority of the recombinant protein was present in the form of inclusion bodies (Figure 7; lanes 2–4), whereas its expression in the soluble fraction was low (Figure 7; lanes 5–7). These results are consistent with others previously reported in the literature, such as mouse C4ST-1, which had to be expressed in *P. pastoris* due to the null soluble expression in *E. coli* (Zhou et al., 2018). The difficulty in expressing this enzyme in *E. coli* may be due in part to the fact that N-linked oligosaccharides bound to C4ST-1 contribute to the production and stability of the active form of the enzyme (Yusa et al., 2005).

Given the low expression of the enzyme in the soluble fraction, it was decided to use the soluble fraction of the cell-free extract directly for the sulfation reactions of CS-0S without further purification.

### 3.4 Coupling of PAPS generation and sulfation modules

**FIGURE 8:**
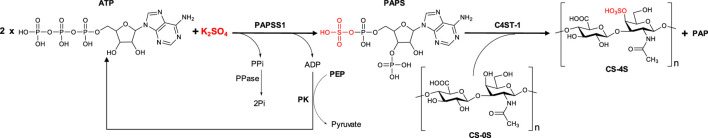
Schematic representation of the biocatalytic cascade developed for the sulfation of unsulfated chondroitin. The cascade involves the regeneration of one of the two ATP molecules used for the *in situ* synthesis of PAPS.

Once both the PAPS generation module with the regeneration system of one of the ATP molecules used in its synthesis and the enzyme C4ST-1 responsible for catalyzing the regioselective sulfation of position 4 of the *N*-acetylglucosamine unit (sulfation module) were available, the two modules were coupled as shown schematically in Figure 8. In this way, the PAPS generated by the PAPSS1 enzyme from ATP and inorganic sulfate can be used *in situ* by the C4ST-1 enzyme to perform the sulfation of chondroitin in a single step without the need for purification of the reaction intermediates.

After purification of the reaction product by dialysis followed by freeze-drying, it was analyzed by NMR. Analysis of disaccharides was discarded because a complete structural characterization of various chondroitin sulfates, including CS-0S and CS-4S, had already been performed in our previous work (Benito-Arenas et al., 2018). This characterization included determination of the composition of the polysaccharides by enzymatic digestion with chondroitin lyase ABC, followed by HPLC analysis and comparison with authentic samples. In addition, preparative HPLC analysis was performed coupled with mass spectroscopy detection. Finally, the samples were analyzed by NMR spectroscopy (^1^H and ^13^C NMR spectra). In this way, we had authentic and confirmed samples of both CS-0S and CS-4S with which to compare the results of our NMR experiments. Under these conditions, the agreement of the chemical shifts of our samples with those of the authentic samples described in our previous work serves to confirm unequivocally the identity of the products of the sulfation reaction described in our manuscript (Table 1). Thus, two compounds were identified, the minor compound corresponding to chondroitin sulfated in position 4 of *N*-acetylglucosamine (CS-4S).

**TABLE 1:** Comparison of the chemical shifts of the obtained reaction product and those of the previously described CS-0S (Benito-Arenas et al., 2018).

	Mayor product^a^		CS-0S^b^			Minor product^a^		CS-4S^b^	
	^1^H-NMR (δ, ppm)	^13^C-NMR (δ, ppm)	^1^H-NMR (δ, ppm)	^13^C-NMR (δ, ppm)		^1^H-NMR (δ, ppm)	^13^C-NMR (δ, ppm)	^1^H-NMR (δ, ppm)	^13^C-NMR (δ, ppm)
U-2	3.35	75.6	3.37	75.2	U-2	—	—	3.42	75.1
U-3	3.57	77.2	3.62	76.8	U-3	—	—	3.65	76.4
N-5	3.68	78.2	3.68	78.0	N-5	—	—	3.89	77.7
U-5	3.69	79.2	3.89	77.3	U-5	—	—	3.74	79.5
U-4	3.74	82.9	3.78	83.0	U-4	—	—	3.84	83.2
N-6	3.75	64.1	3.74	64.0	N-6	—	—	3.83	63.9
N-3	3.80	83.4	3.81	83.2	N-3	—	—	4.07	78.5
N-2	3.94	54.1	3.98	54.2	N-2	—	—	4.08	54.4
N-4	4.11	70.8	4.08	70.8	N-4	4.77	77.3	4.81	79.3
U-1	4.48	107.3	4.53	107.4	U-1	—	—	4.54	106.6
N-1	4.50	104.1	4.49	104.3	N-1	—	—	4.62	103.7

^a^ This work.

^b^ Benito-Arenas et al.(2018).

U, glucuronic acid subunit; N, *N*-Acetylglucosamine.

**FIGURE 9:**
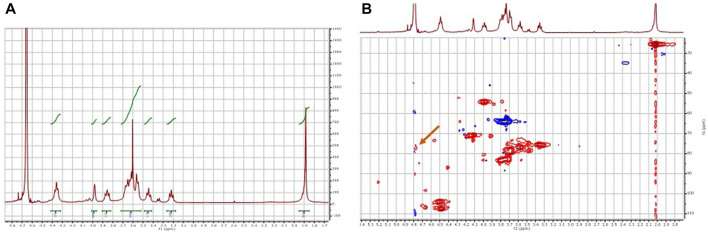
NMR analysis of the product of the biocatalytic sulfation cascade. **(A)** ^1^H-NMR spectrum, **(B)** 2D HSQC spectrum. The arrow in panel **(B)** indicates the crossing signal δ ^1^H = 4.77 δ ^13^C = 77.3 ppm corresponding to the sulfated C4 of *N*-acetylglucosamine.

In the ^1^H-NMR spectrum (Figure 9A), the characteristic signals of the chondroitin disaccharide unit are observed, and the presence of sulfated chondroitin cannot be confirmed. However, if position 4 of the *N*-acetylglucosamine subunit of the disaccharide were sulfated, the signal corresponding to this position would not be screened and would appear below the signal for deuterated water from the solvent. Therefore, it cannot be concluded from the ^1^H-NMR spectrum whether sulfation of the compound has occurred. Therefore, a 2D-NMR HSQC experiment was performed (Figure 9B). In this experiment, a minor correlation (δ ^1^H = 4.77 δ ^13^C = 77.3 ppm) attributable to position 4 of the sulfated *N*-acetylglucosamine subunit was observed, confirming that the developed modular cascade was able to catalyze the regioselective sulfation of chondroitin generating *in situ* the PAPS from ATP and inorganic sulfate.

## 4 Conclusion

For the regioselective sulfation of chondroitin, a biocatalytic cascade was developed that allows PAPS to be obtained *in situ* from ATP and inorganic sulfate, greatly facilitating and lowers the cost of the process. To this end, the human PAPSS1 enzyme and the C4ST-1 sulfotransferase from *R. norvegicus* were heterologously expressed in *E. coli*. While the level of overexpression of PAPSS1 was very satisfactory, the overexpression of the C4ST-1 enzyme needs further optimization. The APS kinase activity of PAPSS1 was coupled to a system for the regeneration of ATP consumed in the phosphorylation of APS. This system has been shown to be necessary for the functioning of the biocatalytic cascade by reducing the inhibition of APS kinase due to the formation of a dead-end enzyme-APS-ADP complex. Once the PAPS generation module and the sulfation module were coupled, sulfation of unsulfated chondroitin could be addressed. NMR analysis of the purified reaction product indicated that the sulfation reaction has taken place, demonstrating the viability of the proposed system.

## Data Availability

The original contributions presented in the study are included in the article/Supplementary Material, further inquiries can be directed to the corresponding author.

## References

[B1] An C. Zhao L. Wei Z. Zhou X. (2017). Chemoenzymatic synthesis of 3′-phosphoadenosine-5′-phosphosulfate coupling with an ATP regeneration system. Appl. Microbiol. Biotechnol. 101, 7535–7544. 10.1007/s00253-017-8511-2 28920175

[B2] Ayuso-Fernández I. Galmés M. A. Bastida A. García-Junceda E. (2014). Aryl sulfotransferase from haliangium ochraceum: A versatile tool for the sulfation of small molecules. ChemCatChem 6, 1059–1065. 10.1002/cctc.201300853

[B3] Bao F. Yan H. Sun H. Yang P. Liu G. Zhou X. (2015). Hydrolysis of by-product adenosine diphosphate from 3′-phosphoadenosine-5′-phosphosulfate preparation using Nudix hydrolase NudJ. Appl. Microbiol. Biotechnol. 99, 10771–10778. 10.1007/s00253-015-6911-8 26293337

[B4] Benito-Arenas R. Doncel-Pérez E. Fernández-Gutiérrez M. Garrido L. García-Junceda E. Revuelta J. (2018). A holistic approach to unravelling chondroitin sulfation: Correlations between surface charge, structure and binding to growth factors. Carbohydr. Polym. 202, 211–218. 10.1016/j.carbpol.2018.08.120 30286994

[B5] Bishop J. R. Schuksz M. Esko J. D. (2007). Heparan sulphate proteoglycans fine-tune mammalian physiology. Nature 446, 1030–1037. 10.1038/nature05817 17460664

[B6] Borges-Walmsley M. I. Turner G. Bailey A. M. Brown J. Lehmbeck J. Clausen I. G. (1995). Isolation and characterisation of genes for sulphate activation and reduction in Aspergillus nidulans: Implications for evolution of an allosteric control region by gene duplication. Mol. Gen. Genet. MGG 247, 423–429. 10.1007/BF00293143 7770049

[B7] Bülow H. E. Tjoe N. Townley R. A. Didiano D. van Kuppevelt T. H. Hobert O. (2008). Extracellular sugar modifications provide instructive and cell-specific information for axon-guidance choices. Curr. Biol. 18, 1978–1985. 10.1016/j.cub.2008.11.023 19062279 PMC2765105

[B8] Burkart M. D. Izumi M. Chapman E. Lin C.-H. Wong C.-H. (2000). Regeneration of PAPS for the enzymatic synthesis of sulfated oligosaccharides. J. Org. Chem. 65, 5565–5574. 10.1021/jo000266o 10970295

[B9] Burkart M. D. Izumi M. Wong C.-H. H. (1999). Enzymatic regeneration of 3′-phosphoadenosine-5′-phosphosulfate using aryl sulfotransferase for the preparative enzymatic synthesis of sulfated carbohydrates. Angew. Chem. Int. Ed. 38, 2747–2750. 10.1002/(sici)1521-3773(19990917)38:18<2747:aid-anie2747>3.0.co;2-2 10508369

[B10] Chapman E. Best M. D. Hanson S. R. Wong C.-H. (2004). Sulfotransferases: Structure, mechanism, biological activity, inhibition, and synthetic utility. Angew. Chem. Int. Ed. 43, 3526–3548. 10.1002/anie.200300631 15293241

[B11] Chen J. Jones C. L. Liu J. (2007). Using an enzymatic combinatorial approach to identify anticoagulant heparan sulfate structures. Chem. Biol. 14, 986–993. 10.1016/j.chembiol.2007.07.015 17884631 PMC4809194

[B12] Chhabra M. Doherty G. G. See N. W. Gandhi N. S. Ferro V. (2021). From cancer to COVID‐19: A perspective on targeting heparan sulfate‐protein interactions. Chem. Rec. 21, 3087–3101. 10.1002/tcr.202100125 34145723 PMC8441866

[B13] Costa D. S. da Reis R. L. Pashkuleva I. (2017). Sulfation of glycosaminoglycans and its implications in human health and disorders. Annu. Rev. Biomed. Eng. 19, 1–26. 10.1146/annurev-bioeng-071516-044610 28226217

[B14] Datta P. Fu L. He W. Koffas M. A. G. Dordick J. S. Linhardt R. J. (2020). Expression of enzymes for 3′-phosphoadenosine-5′-phosphosulfate (PAPS) biosynthesis and their preparation for PAPS synthesis and regeneration. Appl. Microbiol. Biotechnol. 104, 7067–7078. 10.1007/s00253-020-10709-6 32601738

[B15] Gama C. I. Tully S. E. Sotogaku N. Clark P. M. Rawat M. Vaidehi N. (2006). Sulfation patterns of glycosaminoglycans encode molecular recognition and activity. Nat. Chem. Biol. 2, 467–473. 10.1038/nchembio810 16878128

[B16] García-Junceda E. García-García J. F. Bastida A. Fernández-Mayoralas A. (2004). Enzymes in the synthesis of bioactive compounds. Bioorg. Med. Chem. 12, 1817–1834. 10.1016/j.bmc.2004.01.032 15051051

[B17] Günal S. Hardman R. Kopriva S. Mueller J. W. (2019). Sulfation pathways from red to green. J. Biol. Chem. 294, 12293–12312. 10.1074/jbc.REV119.007422 31270211 PMC6699852

[B18] Harjes S. Bayer P. Scheidig A. J. (2005). The crystal structure of human PAPS synthetase 1 reveals asymmetry in substrate binding. J. Mol. Biol. 347, 623–635. 10.1016/j.jmb.2005.01.005 15755455

[B19] Harjes S. Scheidig A. Bayer P. (2004). Expression, purification and crystallization of human 3′-phosphoadenosine-5′-phosphosulfate synthetase 1. Acta Crystallogr. Sect. D. Biol. Crystallogr. 60, 350–352. 10.1107/S0907444903027628 14747722

[B20] Hayes A. Sugahara K. Farrugia B. Whitelock J. M. Caterson B. Melrose J. (2018). Biodiversity of CS–proteoglycan sulphation motifs: Chemical messenger recognition modules with roles in information transfer, control of cellular behaviour and tissue morphogenesis. Biochem. J. 475, 587–620. 10.1042/bcj20170820 29439148

[B21] He W. Zhu Y. Shirke A. Sun X. Liu J. Gross R. A. (2017). Expression of chondroitin-4-O-sulfotransferase in *Escherichia coli* and Pichia pastoris. Appl. Microbiol. Biotechnol. 101, 6919–6928. 10.1007/s00253-017-8411-5 28761999

[B22] Holt C. E. Dickson B. J. (2005). Sugar codes for axons? Neuron 46, 169–172. 10.1016/j.neuron.2005.03.021 15848796 PMC3687205

[B23] Lansdon E. B. Fisher A. J. Segel I. H. (2004). Human 3‘-phosphoadenosine 5‘-phosphosulfate synthetase (isoform 1, brain): Kinetic properties of the adenosine triphosphate sulfurylase and adenosine 5‘-phosphosulfate kinase domains. Biochemistry 43, 4356–4365. 10.1021/bi049827m 15065880

[B24] Leustek T. Murillo M. Cervantes M. (1994). Cloning of a cDNA encoding ATP sulfurylase from *Arabidopsis thaliana* by functional expression in *Saccharomyces cerevisiae* . Plant Physiol. 105, 897–902. 10.1104/pp.105.3.897 8058839 PMC160738

[B25] Leyh T. S. (1993). The physical biochemistry and molecular genetics of sulfate activation. Crit. Rev. Biochem. Mol. Biol. 28, 515–542. 10.3109/10409239309085137 8299360

[B26] Lin C.-H. Shen G.-J. Garcia-Junceda E. Wong C.-H. (1995). Enzymic synthesis and regeneration of 3'-phosphoadenosine 5'-phosphosulfate (PAPS) for regioselective sulfation of oligosaccharides. J. Am. Chem. Soc. 117, 8031–8032. 10.1021/ja00135a028

[B27] Lin T.-S. Hsieh C.-H. Kuo C. Juang Y.-P. Hsieh Y. S. Y. Chiang H. (2020). Sulfation pattern of chondroitin sulfate in human osteoarthritis cartilages reveals a lower level of chondroitin-4-sulfate. Carbohydr. Polym. 229, 115496. 10.1016/j.carbpol.2019.115496 31826425

[B28] Mikami T. Kitagawa H. (2017). Sulfated glycosaminoglycans: Their distinct roles in stem cell biology. Glycoconj. J. 34, 725–735. 10.1007/s10719-016-9732-9 27709407

[B29] Mikami T. Mizumoto S. Kago N. Kitagawa H. Sugahara K. (2003). Specificities of three distinct human chondroitin/dermatan N-acetylgalactosamine 4-O-sulfotransferases demonstrated using partially desulfated dermatan sulfate as an acceptor. J. Biol. Chem. 278, 36115–36127. 10.1074/jbc.M306044200 12847091

[B30] Mueller J. W. Shafqat N. (2013). Adenosine-5′-phosphosulfate – A multifaceted modulator of bifunctional 3′-phospho-adenosine-5′-phosphosulfate synthases and related enzymes. FEBS J. 280, 3050–3057. 10.1111/febs.12252 23517310 PMC3734648

[B31] Peterson S. Frick A. Liu J. (2009). Design of biologically active heparan sulfate and heparin using an enzyme-based approach. Nat. Prod. Rep. 26, 610–627. 10.1039/b803795g 19387498

[B32] Sakamoto K. Ozaki T. Ko Y.-C. Tsai C.-F. Gong Y. Morozumi M. (2019). Glycan sulfation patterns define autophagy flux at axon tip via PTPRσ-cortactin axis. Nat. Chem. Biol. 15, 699–709. 10.1038/s41589-019-0274-x 31061498

[B33] Sambrook J. Fritsch E. F. Maniatis T. (1989). Molecular cloning: A laboratory manual. 2nd ed. New York, NY: Cold Spring Harbour.

[B34] Sekulic N. Dietrich K. Paarmann I. Ort S. Konrad M. Lavie A. (2007). Elucidation of the active conformation of the APS-kinase domain of human PAPS synthetase 1. J. Mol. Biol. 367, 488–500. 10.1016/j.jmb.2007.01.025 17276460 PMC1941671

[B35] Simpson L. S. Widlanski T. S. (2006). A comprehensive approach to the synthesis of sulfate esters. J. Am. Chem. Soc. 128, 1605–1610. 10.1021/ja056086j 16448132

[B36] Venkatachalam K. V. Akita H. Strott C. A. (1998). Molecular cloning, expression, and characterization of human bifunctional 3′-phosphoadenosine 5′-phosphosulfate synthase and its functional domains. J. Biol. Chem. 273, 19311–19320. 10.1074/jbc.273.30.19311 9668121

[B37] Wang T. Liu L. Voglmeir J. (2020). Chemoenzymatic synthesis of ultralow and low-molecular weight heparins. Biochim. Biophys. Acta - Proteins Proteomics 1868, 140301. 10.1016/j.bbapap.2019.140301 31678194

[B38] Xu D. Moon A. F. Song D. Pedersen L. C. Liu J. (2008). Engineering sulfotransferases to modify heparan sulfate. Nat. Chem. Biol. 4, 200–202. 10.1038/nchembio.66 18223645 PMC2676843

[B39] Xu Y. Cai C. Chandarajoti K. Hsieh P.-H. Li L. Pham T. Q. (2014). Homogeneous low-molecular-weight heparins with reversible anticoagulant activity. Nat. Chem. Biol. 10, 248–250. 10.1038/nchembio.1459 24561662 PMC4393012

[B40] Xu Y. Masuko S. Takieddin M. Xu H. Liu R. Jing J. (2011). Chemoenzymatic synthesis of homogeneous ultralow molecular weight heparins. Sci. (80-. ) 334, 498–501. 10.1126/science.1207478 PMC3425363 22034431

[B41] Yamauchi S. Hirahara Y. Usui H. Takeda Y. Hoshino M. Fukuta M. (1999). Purification and characterization of chondroitin 4-sulfotransferase from the culture medium of a rat chondrosarcoma cell line. J. Biol. Chem. 274, 2456–2463. 10.1074/jbc.274.4.2456 9891016

[B42] Yanagisawa K. Sakakibara Y. Suiko M. Takami Y. Nakayama T. Nakajima H. (1998). cDNA cloning, expression, and characterization of the human bifunctional ATP sulfurylase/adenosine 5′-phosphosulfate kinase enzyme. Biosci. Biotechnol. Biochem. 62, 1037–1040. 10.1271/bbb.62.1037 9648242

[B43] Yusa A. Kitajima K. Habuchi O. (2005). N-linked oligosaccharides are required to produce and stabilize the active form of chondroitin 4-sulphotransferase-1. Biochem. J. 388, 115–121. 10.1042/BJ20041573 15628971 PMC1186699

[B44] Zhou X. Chandarajoti K. Pham T. Q. Liu R. Liu J. (2011). Expression of heparan sulfate sulfotransferases in Kluyveromyces lactis and preparation of 3′-phosphoadenosine-5′-phosphosulfate. Glycobiology 21, 771–780. 10.1093/glycob/cwr001 21224284 PMC3091527

[B45] Zhou Z. Li Q. Huang H. Wang H. Wang Y. Du G. (2018). A microbial-enzymatic strategy for producing chondroitin sulfate glycosaminoglycans. Biotechnol. Bioeng. 115, 1561–1570. 10.1002/bit.26577 29484646

