# Supplementary material for: Design of a biocatalytic cascade for the enzymatic sulfation of unsulfated chondroitin with in situ generation of PAPS
Source: Front Bioeng Biotechnol. 2023 Jan 16;11:1099924. doi: 10.3389/fbioe.2023.1099924 (PMC9885120; doi:10.3389/fbioe.2023.1099924)
Supplement: Supplementary file 1 [file DataSheet1.docx]

**Supplementary Material**

**Design of a biocatalytic cascade for the enzymatic sulfation of unsulfated chondroitin with in situ generation of PAPS**

Dianelis T. Monterrey^1†^, Raúl Benito-Arenas^1^, Julia Revuelta^1^, Eduardo García-Junceda^1*^,


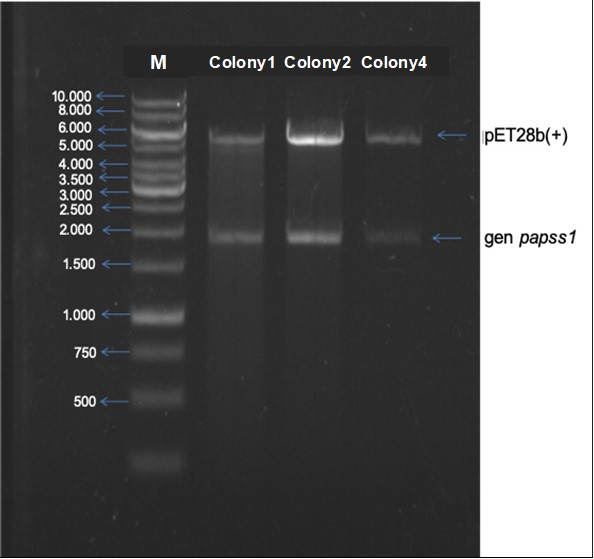


**Figure S1**. Agarose gel of restriction analysis of plasmid pET-28b(+)-*papss1*, purified from three different colonies. M = molecular weight marker.

**Figure S2**. Chromatogram of ATP (A) and ADP (B) standards.

**Figure S3**. Agarose gel of restriction analysis of plasmid pET-TrxA-*chst11*, purified from three different colonies. MWM = molecular weight marker.
